# Ara h 7 isoforms share many linear epitopes: Are 3D epitopes crucial to elucidate divergent abilities?

**DOI:** 10.1111/cea.13496

**Published:** 2019-10-06

**Authors:** Anna M. Ehlers, Marco Klinge, Waltraud Suer, Yvonne Weimann, André C. Knulst, Frithjof Besa, Thuy‐My Le, Henny G. Otten

**Affiliations:** ^1^ Laboratory of Translational Immunology University Medical Center Utrecht Utrecht University Utrecht The Netherlands; ^2^ Department of Dermatology and Allergology University Medical Center Utrecht Utrecht University Utrecht The Netherlands; ^3^ EUROIMMUN AG Lübeck Germany

**Keywords:** 2S albumins, Ara h 7 isoforms, food allergy, linear epitopes, peanut

## Abstract

**Background:**

The peanut allergens Ara h 2, h 6, and h 7 are potent allergens and can trigger severe reactions. Ara h 7 consists of three isoforms differing in their ability to induce basophil degranulation, whereas the ability of Ara h 7.0201 is comparable to Ara h 2 and 6 as shown in previous literature.

**Objective:**

To identify linear epitopes of Ara h 7.0101, Ara h 7.0201 and Ara h 7.0301 recognized by IgE and IgG4 from patients sensitized to Ara h 7 and to investigate their potential to elucidate divergent abilities of the Ara h 7 isoforms in inducing basophil activation.

**Methods:**

Linear epitopes recognized by IgE and IgG4 were mapped by peptide microarray analysis containing 15‐mer peptides of Ara h 2.0201, 6, 7.0101, 7.0201 and 7.0301 and 39 peanut allergic patients sensitized to Ara h 7 (discovery). For validation, 20‐mer peptides containing the minimal epitope and surrounding amino acids were incubated with 25 sensitized patients and 10 controls (validation).

**Results:**

Three out of 14 linear epitopes were unique for each isoform (Ara h 7.0101: aa 97‐109; Ara h 7.0201: aa 122‐133; Ara h 7.0301: aa 65‐74) but scarcely recognized by IgE. The main linear IgE epitope (aa 51‐57) located in the long flexible loop of all Ara h 7 isoforms was bound by antibodies from 31% of the patients (discovery and validation cohort). Regarding IgG4, 55% of the patients recognized an epitope present on all isoforms (aa 55‐65), whereas epitope aa 129‐137, only present on Ara h 7.0101/0.0301, was recognized by 38% of the patients. Recognition was highly individual, although 20% of the patients recognized any linear epitope neither by IgE nor by IgG4 despite a low mean z‐score of ≥ 1.7. Remarkably, only 50% of the patients recognized one or more epitopes by IgE.

**Conclusion & Clinical Relevance:**

Ara h 7 isoforms share many linear epitopes being easily accessible for antibody binding. Unique epitopes, essential to elucidate divergent potencies, were scarcely recognized, suggesting a crucial involvement of conformational epitopes.

## 1 INTRODUCTION

Peanut allergy is one of the most prevalent food allergies worldwide and is in Western and Central Europe often triggered by Ara h 8, a birch pollen‐related PR‐10 protein, or seed storage proteins like Ara h 2 and 6 from the 2S albumin family. While sensitization to Ara h 8 often results in mild reactions such as oral allergy syndrome, Ara h 2 and 6 can induce severe reactions, including anaphylaxis.^1^, ^2^ Ara h 7 is the third member of the 2S albumin family, but it is far less abundant in peanut than Ara h 2 and 6 and shares a sequence homology of 51% and 61%, respectively.^3^

For diagnosing peanut allergy, IgE antibodies against Ara h 2 and Ara h 6 are known to have a very high positive predictive value.^4^, ^5^ Recently, IgE‐binding to Ara h 7 showed a discriminative ability comparable to Ara h 2 and Ara h 6 and this 2S albumin was as potent as Ara h 2 and h 6 in inducing basophil degranulation.^6^ For Ara h 7, three different isoforms—Ara h 7.0101, Ara h 7.0201, Ara h 7.0301—have been described whilst Ara h 7.0101 has only be detected on cDNA but not on protein level.^7^ Ara h 7.0201 was the most potent isoform to induce degranulation. IgE‐binding to this allergen cannot be fully inhibited by Ara h 7.0101, although IgE‐binding to Ara h 7.0101 was completely inhibited by Ara h 7.0201. This data suggest the presence of unique epitopes on Ara h 7.0201.^8^ By amino acid sequence comparisons, these unique epitopes might be located on the distinctive C‐terminus or created by amino acid substitutions within the flexible loops that are sensitive to pepsin or trypsin.^6^ The aim of the study was to define linear epitopes of Ara h 7 isoforms. To this end, we performed a linear epitope mapping using the peptide microarray technique and applying sera from allergic and tolerant patients sensitized to Ara h 7.

## 2 METHODS

### 2.1 Patient selection

For identifying linear epitopes, sera with specific IgE to peanut extract (ImmunoCAP) were screened by EUROLINE (EL, Euroimmun AG, Lübeck, Germany) for sIgE‐binding to heterologously expressed Ara h 7.0201. Overall, 39 sera with sIgE levels ≥16 intensity units (EL) for Ara h 7.0201 were applied for the peptide microarray analysis, and peanut allergy was confirmed by food challenge according to the international consensus protocol or experienced physician diagnosis (discovery cohort).^9^ The cut‐off level was chosen to guarantee the detection of a broad epitope spectrum. In the next phase, the identified epitopes were validated by 25 DBPCFC‐confirmed peanut allergic (n = 22) or tolerant (n = 3) patients with sIgE levels ≥3 intensity units for Ara h 7.0201 (validation cohort).^8^, ^9^ As control, 10 sera without sIgE to Ara h 7.0201 were used. In Table 1, the comparison between CAP‐classes, concentrations and EUROLINE intensity units is shown.^10^ Ethical approval was acquired from the biobank committee of the University Medical Center Utrecht, number 18‐428.

### 2.2 *Peptide chip design*

**Table 1:** Intensity level and corresponding ImmunoCAP classes

CAP‐Class	0	1	2	3	4	5	6
Intensity level	0‐2	3‐6	7‐15	16‐30	31‐50	51‐100	>100
ImmunoCAP [kU/L]	0‐<0.35	0.35‐<0.7	0.7‐<3.5	3.5‐<17.5	17.5‐<50	50‐<100	≥100

For the discovery phase, a peptide microarray with overlapping 15‐mer peptides was commercially obtained (PEPperPRINT), comprising the sequences of Ara h7.0101, Ara h 7.0201 Ara h 7.0301 (offset: 1), Ara h 2.0101, and Ara h 6.0101 (offset: 2). According to the experiences of PEPperPRINT, 15‐mer peptides have the optimal length to provide sufficient sensitivity without significant induction of secondary structures. All peptides were printed as triplicates with a 3 amino acids linker (2x β‐alanine and 1x aspartic acid) to prevent binding of negatively charged fluorescent dyes to positively charged amino acids close to the array surface. For validating the discovered minimal epitopes, a new microarray layout was applied. For each epitope, a 20‐mer peptide was designed containing the minimal epitope and surrounding amino acids, replenished with glycine and serine amino acids.

### 2.3 Microarray incubation

All dilutions and washing steps were performed in working strength universal buffer (EUROIMMUN, purchase order number ZW1100). Patient samples were diluted 1:4 and incubated on the microarrays at 4°C overnight. For the detection of bound IgE and IgG4 antibodies, the arrays were incubated with biotinylated anti‐IgE antibody (clone MHE‐18 1:5000, BioLegend) and biotin anti‐human IgG4 coupled with Neutravidin DyLight 680 (clone HP6025, 1:5000, Southern Biotech) for one hour at room temperature. After washing, the arrays were incubated for one hour at room temperature with fluorescent Neutravidin DyLight 800 (Thermo Fisher), diluted 1:5000 for IgE detection. After washing with dipping buffer (1 mmol/L Tris‐HCl pH 7.4), the peptide microarray slides were dried and scanned with a Licor Odyssey Imager at a wavelength of 800 nm (intensity: 10). Image focus was set to 0.8 mm, and an image resolution of 21 µm was chosen.

### 2.4 Microarray evaluation

Fluorescent signals were extracted using PepSlide Analyzer Software (SICASYS) and exported to CSV files. For data evaluation, logarithmic signal‐noise‐ratios (S) were calculated for each peptide according to:

$$\text{Si}=\log_{2}\frac{\text{Total Fluorescence}\left(\text{Peptide}\right)}{\text{Background Fluoresence}\left(\text{Peptide}\right)}$$

These S‐values were normalized against the S‐values of blank spots on the array, resulting in a z‐score defined as:

$$\text{Zi}=\frac{\text{Si}\;-\text{Median}\left(S\;\text{Blank}\right)}{\text{Mean Deviation}\;\left(S\;\text{Blank}\right)}$$

Significance levels of positive peptide binding were defined based on z‐scores as followed: Z > 1.7 (*P* < .05;*); Z > 2.4 (*P* < .01;**); Z > 3.0 (*P* < .001;***); Z > 4.0 (*P* < .0001). Peptides were only considered if the coefficient of variation for the triplicate of each peptide was lower than 50%. Recognized peptides were defined as epitopes if 3‐5 contiguous peptides with a mean z‐score ≥ 1.7 were detected.

### 2.5 Determination sIgE and sIgG4 sensitization

Specific IgE and IgG4 sensitization to the full‐length protein Ara h 7 was assessed by line blots (EUROIMMUN) according to manufacturer's instructions. Briefly, sera were applied (1:11 for IgE, 1:51 for IgG4) overnight, and after washing three times with universal buffer, bound IgE was detected by an anti‐human IgE and anti‐human IgG_4_‐antibody labelled with alkaline phosphatase. Visualization was provided by adding nitro‐blue tetrazolium/5‐bromo‐4‐chloro‐3’‐indolyphosphate substrate for ten minutes after washing three times with universal buffer.

### 2.6 Modelling 3D structure of Ara h 7 isoforms

3D structure of Ara h 7.0201 was assessed by Modeller software.^11^ Since the amino acid sequences of 2S albumins differ, homology modelling with multiple input sequences and crystal or NMR structures was chosen (Ara h 2 reference 3OB4, Ara h 6 reference 1W2Q, Ric c 3 reference 1PSY, sunflower 2S albumin reference 1S6D, rapeseed 2S albumin reference 1SM7). Five resulting models were evaluated by DOPE score, and the model with the lowest score was selected (DOPE score = −11083.618).

### 2.7 Model assessment

To evaluate the 3D model, the DOPE score per residue was assessed. DOPE scores > −0.3 indicate levels of relatively high energy, pointing towards structural errors. Additionally, the model was applied to the ModFOLD6 server^12^, ^13^ calculating a residue error plot, a global model quality score (0.5348), and a *P*‐value (*P* < .0001). Overall, the structure was of high quality, apart from the flexible loops which are also experimentally difficult to assess. The quality assessment is shown in the Supplementary (Figure S1).

### 2.8 Data analysis

The baseline data were statistically analysed using one‐way ANOVA or Mann‐Whitney *U* test for continuous data and Fisher's exact test for categorical data. Statistical evaluation was performed with GraphPad Prism 7 (GraphPad Software) and SPSS Statistics 21 (IBM Corporation). *P* values ≤ .05 were considered as statistically significant.

## 3 RESULTS

### 3.1 Unique linear IgE epitopes of Ara h 7 isoforms were marginally recognized

**Figure 1:**
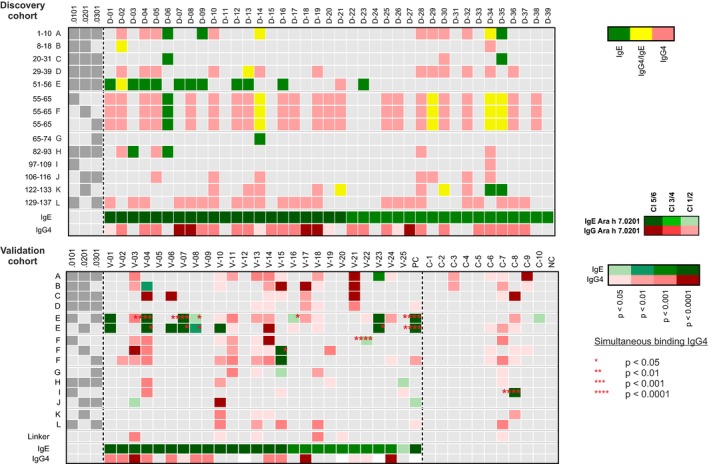
Individual epitope recognition patterns for IgE (green), for IgG4 (red), and for co‐recognition by IgE and IgG4 (yellow); on the left‐hand side, dark grey dots indicate the respective isoform(s) for each epitope. The relative IgE and IgG4 sensitization to Ara h 7 is indicated with different gradation of green (IgE) and red (IgG4) in the bottom of each heatmap. A, Discovery cohort selected by sIgE levels ≥16 intensity units for Ara h 7.0201, the numbers on the left‐hand side indicate the different minimal epitopes identified. B, Validation cohort selected by sIgE levels ≥3 intensity units for Ara h 7.0201 and controls without sIgE to Ara h 7.0201; for validation purposes, peptides containing the minimal epitope and surrounding amino acids were used resulting occasionally in more than one peptide containing the same minimal epitope (eg epitope E). Epitopes recognized by IgE and IgG4 are highlighted based on their significance level (* is *P* < .05, ** is *P* < .01, *** is *P* < .001, **** is *P* < .0001)

To define unique epitopes of Ara h 7 isoforms, we mapped linear epitopes of these proteins by peptide chip analysis using patients’ sera with IgE levels for Ara h 7 ≥ 16 intensity units (corresponding to CAP‐class > 2). Patient characteristics are shown in Table 2. Overall, 14 different linear amino acid sequences (A‐L) were bound by IgE (green) or IgG4 (red) as shown in Figure 1A. Epitope codes are listed in Table 3. Epitope E affiliating to all isoforms was recognized by most patients with a frequency of 31% for IgE and 5% for IgG4 in the discovery cohort. Whilst epitope E was predominately recognized by IgE, epitope F and L were mainly bound by IgG4 (61.5% and 54%). Contrary, the unique epitope G (Ara h 7.0301) showed an IgE recognition frequency of only 2.5% and epitope I (Ara h 7.0101) was not recognized by IgE at all. These unique epitopes were present in the core of the 3D structure of the proteins, and theoretically, they would be only accessible after enzymatic digestion by pepsin or trypsin. In contrast, the unique epitope K (Ara h 7.0201) is located on the flexible C‐terminus and was recognized by IgE from 10% of the included patients. However, this epitope was only recognized by IgG4 (16%) in the validation cohort (Figure 1B). Overall, epitope E was the main IgE epitope in the discovery and validation cohort.

**Table 2:** Patient characteristics and sensitization data

	Discovery cohort (n = 39)	Validation cohort (n = 25)	Control group (n = 10)	*P*‐value
Age (median [IQR])	25 [18‐54]	23 [18‐38]	39 [20‐66]	.001^d^
Sex female [n, %]	21 (54%)	8 (32%)	7 (70%)	.084
Food challenge [n, %]	6 (15%)	25 (100%)	10 (100%)	<.0001^d^
Symptoms [n, %]				
Objective	25 [64%]	14 [56%]		.341
Subjective	13 [33%]	7 [28%]		
No symptoms	NA	3 [12%]	8 [80%]^a^	
Sensitization				
ImmunoCAP peanut extract	19 [1.2‐73 kU/l]	11.2 [0.62‐100 kU/l]	0.4 [0‐9.35 kU/l]^b^	.378
EUROLINE Ara h 2^c^	30 [1‐98 EL‐int.]	71 [1‐ >100 EL‐int.]	<3 [0‐2 EL‐int.]	.008
EUROLINE Ara h 6^c^	58 [4‐ >100 EL‐int.]	56 [2‐ >100 EL‐int.]	<3 [0‐1 EL‐int.]	.992
EUROLINE Ara h 7^c^	61 [16‐ >100 EL‐int.]	98 [4‐ >100 EL‐int.]	<3 [0‐1 EL‐int.]	.084

a Two provocation were inconclusive.

b Data from n = 6.

c EUROLINE intensities (EL‐int.): <3 ≙ EAST‐class 0; 3‐6 ≙ EAST‐class 1; 7‐15 ≙ EAST‐class 2; 16‐30 ≙ EAST‐class 3; 31‐50 ≙ EAST‐class 4; 51‐100 ≙ EAST‐class 5; >100 ≙ EAST‐class 6.

d Significant difference between discovery or validation cohort and control group (age); significant difference between discovery cohort and validation cohort or control group (food challenge).

**Table 3:** Linear epitopes of Ara h 7 isoforms

Epitope	Residues (without signal sequence)	aa sequence	Isoform	Specificity
A	1‐9	TRWDPDRGSR	all	IgE/IgG4
B	8‐18	GSRGSRWDAPS	0.0101, 0.0201	IgE/IgG4
C	20‐31	DDQCQRQIQRA	all	IgE/IgG4
D	29‐39	QRANLRPCEEH	all	IgE/IgG4
E	51‐57	EQDEYPY	all	IgE/IgG4
F	55‐65	YPYSRRGSRGR	0.0101	IgE/IgG4
	55‐65	YPYIQRGSRGQ	0.0201	IgE/IgG4
	55‐65	YPYSQRGSRGR	0.0301	IgE/IgG4
G	65‐74	RRPGESDEDQ	0.0301	IgE
H	82‐93	LNRFQNNQRCMC	all	IgE/IgG4
I	97‐109	QQILQNQSFWVPA	0.0101	IgG4
J	106‐116	RFQQDRSQLHQ	0.0201, 0.0301	IgG4
K	122‐133	NLPQNCGFRSPS	0.0201	IgE/IgG4
L	129‐137	RVQVTKPLR	0.0101, 0.0301	IgG4

### 3.2 Recognition patterns of linear epitopes were highly individual

All patients showed individual linear epitope recognition patterns as shown in Figure 1A,B. Two patients (D‐06 and D‐35) recognized up to four different epitopes by IgE in the discovery cohort. Occasionally, epitopes, particularly epitope E and F, were bound simultaneously by patients IgE and IgG4 antibodies (yellow). Moreover, epitopes bound by IgG4 were located in the neighbourhood of an epitope recognized by IgE in eight patients. For example, epitope E was bound by IgE and epitopes D and F were bound by IgG4 in patient D‐04. Certainly, half of the patients did not show any sIgE binding to linear peptides whilst all of them recognized the complete allergens, and ten of them showed at least IgG4‐binding. This suggests the importance of conformational epitopes to elucidate strong IgE‐binding to the full‐length protein. Importance of conformational epitopes was supported by IgE‐binding to even fewer linear epitopes in the validation cohort.

### 3.3 Similar epitope recognition resulted in divergent ability to induce basophil degranulation

In a previous study, the ability to induce basophil degranulation was studied for all Ara h 7 isoforms.^6^ Patients D‐23, V‐01, and D‐13 correspond to the patients N07, N12, and N14, respectively, in that study. Even though IgE antibodies from patients D‐23 and V‐01 recognize only epitope E, patient D‐23 having high IgE titres to all full‐length proteins showed overall low degranulation, whereas patient V‐01, also having high IgE titre, showed strong basophil degranulation after stimulation with Ara h 7.0201. Patient D‐13 had a more diverse sIgE recognition profile (epitopes E + D which are present on all Ara h 7 isoforms), and degranulation was induced by Ara h 7.0201 and Ara h 7.0301, although sIgE levels to Ara h 7.0301 were low. These data indicate that IgE recognition patterns of peptides alone cannot elucidate the divergent ability in inducing basophil degranulation.

### 3.4 Amino acid replacements do not necessarily influence binding and recognition

**Figure 2:**
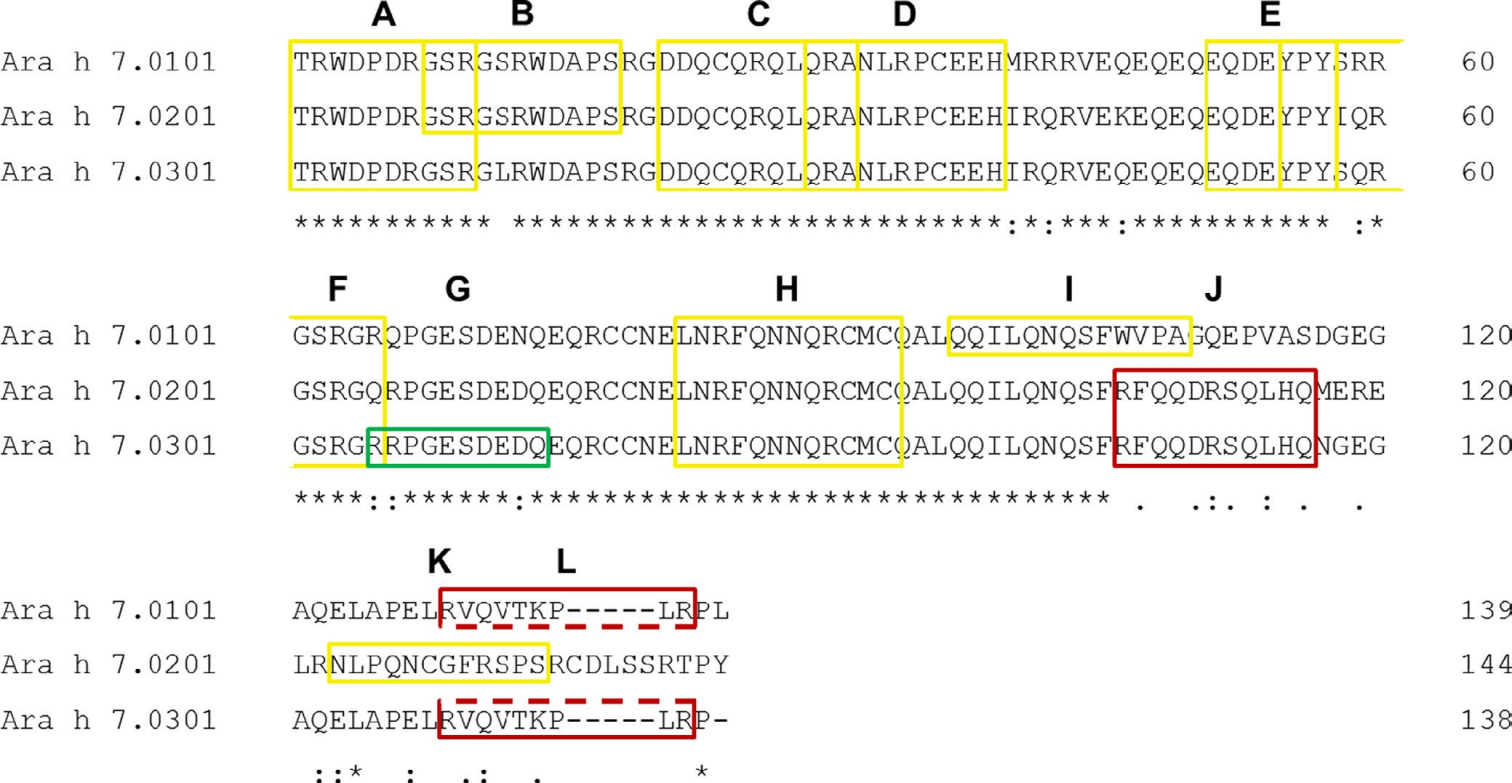
Identified linear epitopes highlighted on the amino acid sequences of Ara h 7.0101, 0.0201 and 0.0301, green: exclusively recognized by IgE; yellow: recognized by IgE and/or IgG4; red: exclusively recognized by IgG4

A complete overview of identified linear epitopes is shown in Figure 2. Interestingly, epitope F showed amino acid differences between all isoforms, but this did not result in divergent minimal epitope recognition in the discovery cohort (Figure 1A). This observation suggested the relevance of residues 55‐57 (Tyr‐Pro‐Tyr) and 61‐63 (Gly‐Ser‐Arg) for antibody binding. Surprisingly, antibody binding of epitope F was not influenced by polar amino acids as glutamine and arginine (aa 59, aa 65). However, surrounding amino acids of epitope F, as considered in the peptide design for the validation cohort, had a great impact on antibody binding (aa 66; V‐02, V‐10, V‐15, V‐19, V‐22, V‐24). Moreover, surrounding residues of epitope E differing between Ara h 7.0101/0.0301 and Ara h 7.0201 (46Lys ‐> Gln and 58Ile ‐> Ser) resulted in divergent antibody binding of the minimal epitope (Figure 1B). IgE antibodies of two patients (V‐06 and V‐10) only recognized the sequence of Ara h 7.0201 and IgG4 antibodies of six patients only the sequence of Ara h 7.0101/0.0301. This indicates an oligoclonal antibody repertoire recognizing the same minimal epitope.

### 3.5 Linear epitopes recognized by IgE are often located on flexible loops

**Figure 3:**
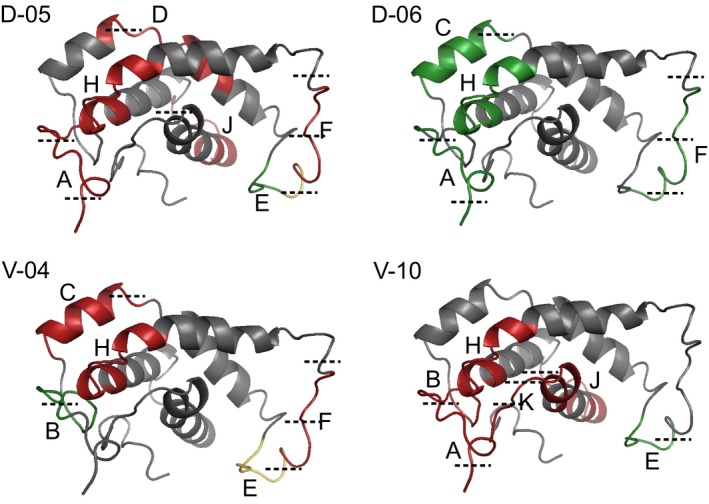
Divergent epitope recognition patterns of four selected patients (D‐05, D‐06, V‐04 and V‐10) were mapped on the 3D model of Ara h 7.0201, and theoretical cutting site were marked with black dash lines. Epitopes recognized by IgE are highlighted in green, and epitopes recognized by IgG4 are highlighted in red

The location of epitopes was shown by applying epitope recognition patterns of four individual patients on the 3D model of Ara h 7.0201 (Figure 3). Epitopes A, B, E, F, K were situated in the flexible loops of the model and epitopes D, H, J in a combination of a flexible loop and an α‐helix. Epitopes A, B, F and K contained multiple theoretical cutting sites for trypsin and pepsin whilst epitope E contained only cutting sites in the beginning and in the end of its amino acid sequence. Cutting sites of epitope E were altered by substitutions of surrounding amino acids. A cutting site for trypsin was introduced on Ara h 7.0201 (aa 46) and a cutting site for pepsin on Ara h 7.0101/0.0301 (aa 56). Contrary, unique linear epitopes of Ara h 7.0101 and 0.0301 were in the core, not accessible without enzymatic digestion. Due to less disulphide bridges, these isoforms are probably less resistant against enzymatic digestion, making these amino acid sequences more easily accessible for Ara h 7.0101/0.0301 than for Ara h 7.0201.

## 4 DISCUSSION

Divergent abilities of Ara h 7 isoforms in inducing basophil degranulation suggested the presence of unique epitopes for Ara h 7.0201 as compared to the other isoforms. This hypothesis was supported by inhibition assays showing complete inhibition of IgE‐binding to Ara h 7.0101 by Ara h 7.0201, although IgE‐binding to Ara h 7.0201 was not fully inhibited by Ara h 7.0101.^6^, ^8^ These observations can partially be elucidated by the newly mapped linear epitopes in this study. Epitope E was predominately recognized by IgE in 31% of the patients whilst epitope F was immunodominant regarding IgG4 binding (55%). Nevertheless, unique epitopes of all isoforms were scarcely and linear epitopes in general were infrequently recognized despite the relatively low threshold of a mean z‐score of ≥1.7. Since the absence of epitope recognition by IgE was not associated with lower sIgE levels to Ara h 7 (Figure 1), lack of sensitivity can be excluded. Thus, our results suggest the importance of conformational epitopes and to lesser extent the importance of IgE antibodies with deviant affinities to understand divergent abilities of the Ara h 7 isoforms in inducing basophil degranulation.

Surprisingly, the main linear IgE epitope (E), located in the long flexible loop, was recognized by only 32.5% of the patients. Other epitopes were detected by even less patients. Limited IgE‐binding to linear epitopes was also observed for Ara h 6 and to a fewer extent for Ara h 2. For Ara h 6, seven linear IgE epitopes were identified whereof the main epitope (aa 97‐106 without signal sequence) was recognized by only 30% of the patients, comparable to our data for Ara h 7 (31% for epitope E). Regarding Ara h 2, linear epitopes were recognized by a greater number of patients, varying from 75% to 100% of the included patients depending on the study considered (aa 10‐18 and aa 42‐60 without signal sequence).^14^, ^15^ Contrary to the other two 2S albumins, Ara h 2 is endowed with a very long flexible loop containing the main epitope and five proline residues (cf. 0 for Ara h 6 and 1 for Ara h 7). Proline residues can be modified by Maillard reaction in presence of reducing sugars occurring during the roasting process. This modification increased the allergenicity of Ara h 2 and underlined the importance of proline residues.^16^, ^17^ However, deletion of flexible loops containing the main linear epitopes diminished the sIgE binding only on an individual basis, showing the additive of conformational epitopes to elucidate IgE‐binding to Ara h 2.^16^, ^18^

Recognition of linear Ara h 7 epitopes by individual patients was highly distinct, stretching from multiple to none linear epitopes recognized by IgE. Individual recognition patterns were also observed for Ara h 2 in previously conducted studies.^19^, ^20^ Occasionally, in the present study epitope recognition by IgE was accompanied by IgG4‐binding for the same epitope. Additionally, IgG4 binding was observed in the neighbourhood of an epitope bound by IgE. Consistently with our results, overlapping IgE and IgG4 epitopes for Ara h 1, 2, 3, 6, 8, and 9 were described previously in partly severely reacting peanut allergic patients.^21^ Moreover, amino acids important for antibody binding were consistent for IgE‐ and IgG4‐binding which is in line with our observation for epitope F.^20^ Contrary, amino acid replacements in the surrounding area of epitope E influenced the binding of IgE and IgG4 antibodies. While aa 46 was important for IgG4‐binding in six patients, aa 58 was important for IgE‐binding in two patients, suggesting an oligo‐ or polyclonal response towards one epitope and limited clonal relation between IgE and IgG4 antibodies, at least in some patients. Overall, IgE and IgG4 can bind the same epitope, also simultaneously, although critical amino acids can differ for IgE and IgG4 antibodies.

Linear epitopes were mostly located in the flexible loops of the 3D protein model. Due to these loci, the epitopes are accessible for antibody binding, but also for enzymatic degradation. Mainly epitope E was located on the longest flexible loop of the Ara h 7 isoforms. This is in line with the main epitopes of Ara h 2 and 6 showing similar loci. However, unique epitopes of Ara h 7.0101 and Ara h 7.0301 were found in the α‐helices, making them only accessible after enzymatic digestion.

To validate the applied technique, we additionally mapped linear epitopes of Ara h 2 and 6. Compared with previous literature, the same main epitopes were found, although two epitopes of Ara h 2 and two of Ara h 6 were not detected (Table S1). However, comparing previous literature among each other, the epitopes described were only partly detected by other studies.^14^, ^15^, ^21^

The identification of conformational epitopes might help elucidating the divergent potencies of the Ara h 7 isoforms. Since detecting and characterizing conformational epitopes is more sophisticated than the identification of linear epitopes, especially with polyclonal serum, human monoclonal antibodies might be a suitable tool for the identification of conformational epitopes. Moreover, human monoclonal antibodies directed to one specific epitope can also be characterized for their exact affinity.^22^

In conclusion, recognition of the 14 new mapped linear epitopes belonging to all three Ara h 7 isoforms was highly individual, and epitopes predominately bound by IgG4 varied from epitopes bound by IgE. These recognition patterns scarcely elucidated the divergent potency of Ara h 7 isoforms, indicating the importance of the addition conformational epitopes.

## CONFLICT OF INTEREST

W. Suer and Y. Weimann are employees of EUROIMMUN AG, Lübeck, Germany and M. Klinge and F. Besa are former employees of EUROIMMUN AG, Lübeck, Germany. The research position of A. Ehlers is partially funded by EUROIMMUN AG, Lübeck, Germany. Other authors have no conflicts of interest to declare.

## AUTHOR CONTRIBUTION

AE, MK, WS, YW, FB and HO involved in experimental design; AE, AK and TML involved in patient selection; AE, MK and FB involved in experimental performance; AE and MK collected and analysed the data; AE and MK drafted the manuscript; AE, MK, WS, AC and HO: contributed to data interpretation; WS, YW, FB, AC, TML and HO critically revised the manuscript.

## INFORMED CONSENT STATEMENT

This study was carried out in accordance with the University Medical Center Utrecht, Biobank Regulations, which are in compliance with the applicable national and international laws and regulations. These regulations permit the use of “residual material from diagnostic testing” for research, unless the patient objects (Article 8, “no objection” procedure). None of the included patients objected the use of their serum. The protocol was approved by the Biobank Research Ethics Committee of the University Medical Center Utrecht under the protocol number 18‐428.

## Data Availability

All data generated or analysed during this study are included in this published article and its supplementary information files.
