# Supplementary material for: Ara h 7 isoforms share many linear epitopes: Are 3D epitopes crucial to elucidate divergent abilities?
Source: Clin Exp Allergy. 2019 Oct 6;49(11):1512–9. doi: 10.1111/cea.13496 (PMC6900131; doi:10.1111/cea.13496)
Supplement: Supplementary file 1 [file CEA-49-1512-s001.pdf]

Graphical ModFOLD6 results for Ara h 7.0201				
**Model name**	**Confidence and p-value**	**Global model quality score**	**Residue error plot**	**3D view of residue error**
Ara h 7.0201	CERT: 4.792E-4	0.4604	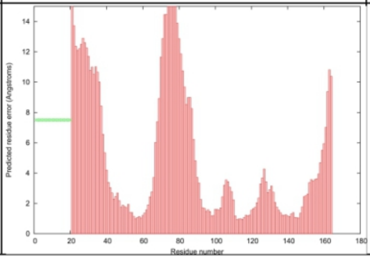	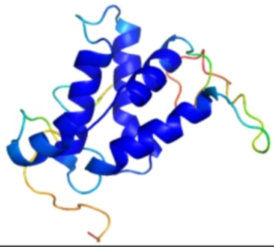

[A]

[B]

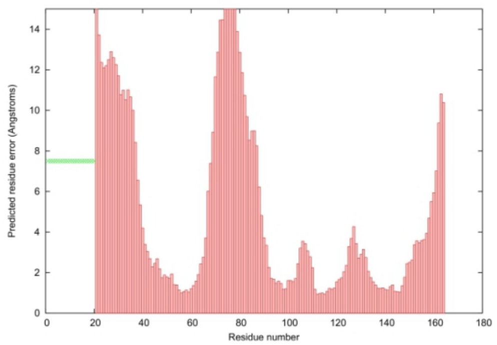

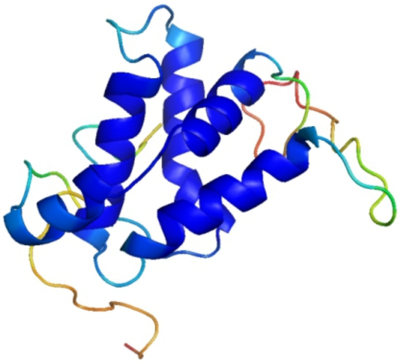

[C]

p-value cut-off	Confidence	Description
p < 0.001	CERT	Less than a 1/1000 chance that the model is incorrect.
p < 0.01	HIGH	Less than a 1/100 chance that the model is incorrect.
p < 0.05	MEDIUM	Less than a 1/20 chance that the model is incorrect.
p < 0.1	LOW	Less than a 1/10 chance that the model is incorrect.
p > 0.1	POOR	Likely to be a poor model with little or no similarity to the native structure

Supplemental Figure 1

The quality of the Ara h 7.0201 3D model was assessed by ModFOLD6 webserver (https://www.reading.ac.uk/bioinf/ModFOLD/).

[A] Error residue plot showing the predicted residue error; the loop regions have a high predicted residue error whilst the Φ-helices have a low predicted residue error. In general, loop regions are hard to predict and to measure since loops are very flexible

[B] Predicted 3D model of Ara h 7.0201 with highlighted residues based on B-factors.

[C] Table overview of quality scores used by the ModFOLD6 webserver
