# Supplementary material for: Ara h 7 isoforms share many linear epitopes: Are 3D epitopes crucial to elucidate divergent abilities?
Source: Clin Exp Allergy. 2019 Oct 6;49(11):1512–9. doi: 10.1111/cea.13496 (PMC6900131; doi:10.1111/cea.13496)
Supplement: Supplementary file 2 [file CEA-49-1512-s002.docx]

**Supplementary Table**

**Table S1:** Validation of peptide chip platform by identification of known Ara h 2 and Ara h 6 epitopes

|  | Peptide | Residues  (without signal sequence) | Publication* | Identified? | Specificity |
| --- | --- | --- | --- | --- | --- |
| Ara h 2.0201 | *HASA*RQQWEL | 1-5 | 1 | x (overlapped) | IgE/IgG4 |
|  | QWELQGDR | 3-10 | 1 |  |  |
|  | DRRCQSQLER | 9-18 | 1, 2, 3 | x | IgG4 |
|  | LRPCEG(Q)HLMQ | 21-30 | 1, 3 | x | IgG4 |
|  | KIQRDEDS | 31-38 | 1 |  |  |
|  | *P*EQHLMQ KIQRDEDS*Y* | 25-30/31-39 | 2 | x | IgG4 |
|  | RDPYSP | 41-47/ 60-65 | 1, 2, 3 | x | IgE/IgG4 |
|  | SQDPYSPS | 47-54 | 1 | x | IgE/IgG4 |
|  | DPYSPSPYDRR | 61-71 | 2, 3 | x | IgE/IgG4 |
|  | RRGAGSSQHQ | 70-79 | 2 | x | IgE |
|  | CNELNEFENNQR | 83-94 | 2, 3 | x | IgE |
|  | CEALQQIMENQSD | 97-109 | 1, 3 |  |  |
|  | QQIMENQ | 101-107 | 1 |  |  |
|  | LQGRQQ*EQQ* | 111-119 | 1, 3 | x | IgG4 |
|  | KRELRNL*PQQ* | 121-130 | 1, 2, 3 | x | IgE/IgG4 |
|  | *CGLRAP*QRCDLEVE | 137-144 | 1, 3 | x | IgE |
|  | EVESGGRDR | 142-150 | 2 |  |  |
| Ara h 6 | MRRERGRGGDSSS | 1-13 | 3 | x | IgE/IgG4 |
|  | SSCERQVDRVNLK | 11-24 | new | x | IgG4 |
|  | KPCEQHIMQRI | 24-34 | 3 | x | IgG4 |
|  | GEQEQ | 36-40 | new | x | IgE |
|  | YDSYDIR | 41-47 | 3 | x | IgE/IgG4 |
|  | CDELNEMENTQR | 59-70 | 3 |  |  |
|  | QQQRCCDELNE | 54-64 | new | x | IgG4 |
|  | CEALQQIMENQCD | 73-85 | 3 | x | IgE |
|  | KRELRMLPQQ | 97-106 | 3 | x | IgE/IgG4 |
|  | CNFRAPQRCDLDV | 107-119 | 3 |  |  |
|  | RCDLDVSGGRC | 114-124 | new | x | IgE/IgG4 |

*****1: Stanley *et al*. 1997; 2: Shreffler *et al.* 2005; 3: Ostu *et al.* 2015
